# Seaweed Supplements Normalise Metabolic, Cardiovascular and Liver Responses in High-Carbohydrate, High-Fat Fed Rats

**DOI:** 10.3390/md13020788

**Published:** 2015-02-02

**Authors:** Senthil Arun Kumar, Marie Magnusson, Leigh C. Ward, Nicholas A. Paul, Lindsay Brown

**Affiliations:** 1 Centre for Systems Biology, University of Southern Queensland, Toowoomba 4350, Australia; E-Mail: senthil.ibt@gmail.com; 2 MACRO—the Centre for Macroalgal Resources and Biotechnology, and College of Marine & Environmental Sciences, James Cook University, Townsville, QLD 4811, Australia; E-Mails: marie.magnusson@jcu.edu.au (M.M.); nicholas.paul@jcu.edu.au (N.A.P.); 3 School of Chemistry and Molecular Biosciences, The University of Queensland, Brisbane 4072, Australia; E-Mail: l.ward@uq.edu.au; 4 School of Health and Wellbeing, The University of Southern Queensland, Toowoomba 4350, Australia

**Keywords:** obesity, hypertension, fatty liver, tropical seaweeds, soluble fibre

## Abstract

Increased seaweed consumption may be linked to the lower incidence of metabolic syndrome in eastern Asia. This study investigated the responses to two tropical green seaweeds, *Ulva ohnoi* (UO) and *Derbesia tenuissima* (DT), in a rat model of human metabolic syndrome. Male Wistar rats (330–340 g) were fed either a corn starch-rich diet or a high-carbohydrate, high-fat diet with 25% fructose in drinking water, for 16 weeks. High-carbohydrate, high-fat diet-fed rats showed the signs of metabolic syndrome leading to abdominal obesity, cardiovascular remodelling and non-alcoholic fatty liver disease. Food was supplemented with 5% dried UO or DT for the final 8 weeks only. UO lowered total final body fat mass by 24%, systolic blood pressure by 29 mmHg, and improved glucose utilisation and insulin sensitivity. In contrast, DT did not change total body fat mass but decreased plasma triglycerides by 38% and total cholesterol by 17%. UO contained 18.1% soluble fibre as part of 40.9% total fibre, and increased magnesium, while DT contained 23.4% total fibre, essentially as insoluble fibre. UO was more effective in reducing metabolic syndrome than DT, possibly due to the increased intake of soluble fibre and magnesium.

## 1. Introduction

Obesity, defined as excess body fat, is a major health-care problem that increases the risk of cardiovascular and metabolic disorders such as hypertension, ischaemic stroke, insulin resistance, impaired glucose tolerance, hyperinsulinaemia and dyslipidaemia [1,2]. Current treatment of obesity is aimed at modifying dietary habits, lowering calorie and fat intake, and increasing exercise to increase calorie expenditure [3,4], rather than drugs. Long-term drug treatment of obesity shows modest effects in many patients, and there are potential health hazards when drug therapies are combined for obesity management [5].

Seaweeds are considered as part of a healthy diet, especially in Japan, Korea, China and the Philippines [6,7]. Seaweeds possess anti-diabetic, antioxidant, anti-obesity, anti-hyperlipidaemic and anti-inflammatory activities [8]. Seaweeds contain higher potassium, magnesium and calcium ion concentrations than other foods [7]. Seaweeds may prevent diet-induced cardiovascular disease as an alternative source of dietary fibre [9]. Fibre is the largest component of the seaweed biomass [10,11] and therefore may be present in sufficient amounts when included in the diet to prevent metabolic syndrome associated with obesity, type 2 diabetes and cardiovascular complications [12]. Seaweeds are typically low in fat (<5%), with the omega-3 fatty acids as the major fat component, including α- and γ-linolenic acids, eicosapentaenoic acid and docosahexaenoic acid [13].

The combination of fibre as polysaccharides, with antioxidants, omega-3 fatty acids and minerals indicates that seaweeds could be targeted as functional foods for metabolic syndrome in westernised diets [6,8]. *Ulva* species showed cholesterol-lowering and cardioprotective properties, as well as anti-inflammatory potential [14,15]. Supplementation of a high-calorie diet with the edible green seaweed *Ulva linza* lowered intra-abdominal fat pads by 35%, blood pressure by 35%, concentrations of blood glucose by 31%, and serum cholesterol and triglycerides concentrations by 17% and 20% [16]. The fast growth rate and broad geographical distribution of tropical species of *Ulva*, such as *Ulva ohnoi* (UO), could provide a sustainable source of these seaweeds for new applications as functional foods as they are suitable for intensive aquaculture production [17]. Another tropical green seaweed with potential for commercialisation is *Derbesia tenuissima* (DT), which has been targeted for its nutritional attributes, especially a higher content of polyunsaturated fatty acids [18]. In contrast to *Ulva*, there is little evidence for *Derbesia* species as a functional food in metabolic syndrome, although methanolic extracts of *Derbesia* species showed high inhibitory activity* in vitro* against protein tyrosine phosphatase 1B [19], a negative regulator of insulin receptors associated with signal transduction.

This study measured the responses to two tropical green seaweeds, *Ulva ohnoi* and *Derbesia tenuissima*, on metabolic parameters and on the structure and function of the cardiovascular system and liver in rats fed a high-carbohydrate, high-fat diet. These rats showed symptoms of metabolic syndrome with metabolic abnormalities, cardiovascular remodelling, and non-alcoholic steatohepatitis [20]. Seaweed supplementation was given for the final 8 weeks of a 16 week protocol. The structure and function of the heart were characterised by echocardiography, isolated Langendorff heart preparation and histopathology, while the structure and function of the liver were characterised by histopathology and plasma biochemical analyses. In addition, metabolic function was characterised by fat measurements, and glucose and insulin tolerance tests. We show that tropical green seaweeds, in particular UO, improve cardiovascular, liver and metabolic parameters in this rat model of human metabolic syndrome.

## 2. Results

### 2.1. Nutritional Composition of Seaweeds

The seaweed supplements differed in their concentrations of soluble and insoluble fibre, fatty acids and minerals. The total dietary fibre content of UO was 40.9% of dry algae with 18.1% as soluble fibre and 22.8% as insoluble fibre while DT contained 23.4% of dry algae, and this was only insoluble fibre. DT had higher fatty acid content (4.9% of dry algae) than UO (1.2% of dry algae), with higher omega-3 fatty acid content (38.8% of total fatty acids) and omega-6 fatty acid content (12.7% of total fatty acids). Mineral contents of UO and DT are given in Supplementary Table S1. The magnesium intake of high-carbohydrate, high-fat + *Ulva ohnoi* 5% (HUO) rats (59.5 mg/day) was higher than high-fat + *Derbesia tenuissima* 5% (HDT) (29.3 mg/day) and H (15.1 mg/day) rats (Supplementary Table S2). Neither UO nor DT altered the total fatty acids intake in corn starch + *Ulva ohnoi* 5% (CUO) and corn starch + *Derbesia tenuissima* 5% (CDT) compared to C rats, nor in HUO and HDT compared to H rats (Supplementary Table S3). The intake of α-linolenic acid was higher in CDT (28.9 mg/day) than in HDT (16.6 mg/day) treated rats. CDT rats had higher eicosapentaenoic acid intake (1.9 mg/day) than HDT rats (1.3 mg/day).

### 2.2. Metabolic Variables

Consumption of food and water was higher in C rats than H rats (Table 1). Compared with C groups (C and CUO), increased energy intake occurred with no change in body weight gain and energy efficiency in H and HUO rats (Table 1). An increased body weight gain was observed in both CDT and HDT rats compared to C rats, while only HDT rats had an increased energy intake (Table 1).

Metabolic parameters are given in Table 2. Compared with C rats, H rats had lower lean mass with higher total body fat mass, abdominal fat mass, abdominal circumference and visceral adiposity index. HUO but not HDT lowered total body fat mass. The glucose utilisation and insulin sensitivity measured as AUC were improved in HUO and HDT rats, compared with H rats. The bone mineral content was higher in H rats, compared with C rats with no effect of either seaweed treatment. Increased plasma concentrations of NEFA, triglycerides and total cholesterol were observed in H rats, whereas no changes were observed in C groups. Plasma NEFA concentrations were increased in HUO rats. DT treatment did not change the plasma NEFA concentration but normalised plasma triglycerides and total cholesterol concentrations in HDT rats. No changes were observed in plasma sodium, potassium and magnesium ion concentrations in the treatment groups.

**Table 1:** Dietary parameters in rats fed C or H and with either *Ulva ohnoi* or *Derbesia tenuissima*.

Variable	C	CUO	CDT	H	HUO	HDT	Diet	Treatment	Interaction
Food intake, g/day	35.7 ± 3.7 ^a^	33.6 ± 3.6 ^a^	32.2 ± 2.4 ^a^	22.0 ± 2.0 ^b^	21.6 ± 0.8 ^b^	21.9 ± 1.3 ^b^	<0.0001	0.78	0.81
Water intake, mL/day	32.0 ± 3.4 ^a^	33.9 ± 2.8 ^a^	33.6 ± 2.4 ^a^	20.0 ± 1.8 ^b^	24.8 ± 0.8 ^b^	20.9 ± 1.7 ^b^	<0.0001	0.37	0.73
Energy intake, kJ/day	396.7 ± 20.2 ^b^	398.9 ± 11.1 ^b^	406.6 ± 11.3 ^b^	469.9 ± 9.3 ^a^	483.2 ± 15.2 ^a^	478.2 ± 4.1 ^a^	<0.0001	0.75	0.86
Feed conversion efficiency, %	2.8 ± 0.3 ^ab^	2.5 ± 0.3 ^b^	4.1 ± 0.4 ^a^	2.9 ± 0.3 ^ab^	2.9 ± 0.2 ^ab^	3.6 ± 0.5 ^ab^	1.00	0.004	0.44
Body weight gain, %	11.1 ± 1.1 ^b^	9.9 ± 1.1 ^b^	16.7 ± 1.5 ^a^	12.8 ± 1.4 ^ab^	13.8 ± 1.2 ^ab^	17.3 ± 2.2 ^a^	0.10	0.001	0.54

Values are mean ± SEM, *n* = 8–10, over the last 8 weeks of the 16 week protocol. Means within a row with unlike superscript differ, *p* < 0.05. C, corn starch fed rats; CUO, corn starch rats treated with *Ulva ohnoi*; CDT, corn starch rats treated with *Derbesia tenuissima*; H, high-carbohydrate, high-fat diet fed rats; HUO, high-carbohydrate, high-fat rats treated with *Ulva ohnoi*; HDT, high-carbohydrate, high-fat rats treated with *Derbesia tenuissima*.

**Table 2:** Metabolic parameters in rats fed C or H and with either *Ulva ohnoi* or *Derbesia tenuissima*.

Variable	C	CUO	CDT	H	HUO	HDT	*P*-Value		
							Diet	Treatment	Interaction
Bone mineral content, g	12.4 ± 0.3 ^c^	12.4 ± 0.5^ c^	13.5 ± 0.4^ bc^	15.1 ± 0.5^ a^	14.3 ± 0.4^ ab^	15.7 ± 0.4^ a^	<0.0001	0.017	0.64
Bone mineral density, g/cm^2^	0.162 ± 0.003	0.163 ± 0.003	0.164 ± 0.003	0.167 ± 0.002	0.161 ± 0.002	0.162 ± 0.002	0.88	0.63	0.30
Total lean mass, g	319.1 ± 10.3 ^ab ^	302.4 ± 2.5 ^bc ^	333.2 ± 6.6^ a^	271.4 ± 8.4 ^c ^	292.3 ± 11.8^ bc ^	284.4 ± 3.9^ c ^	<0.0001	0.23	0.04
Total body fat mass, g	85.5 ± 7.4^ c^	103.8 ± 9.8^ c^	100.1 ± 7.9^ c^	201.6 ± 10.9 ^a^	153.1 ± 14.6 ^b^	190.0 ± 18.9 ^a^	<0.0001	0.32	0.026
Abdominal circumference, cm	19.6 ± 0.4 ^b^	18.8 ± 0.2 ^b^	19.6 ± 0.2 ^b^	22.0 ± 0.4 ^a^	21.4 ± 0.2 ^a^	22.2 ± 0.6 ^a^	<0.0001	0.08	0.95
Glucose AUC, mmol/L/120 min	637.6 ± 8.4 ^c^	674.9 ± 17.6 ^bc^	700.7 ± 22.0 ^b^	809.7 ± 12.9 ^a^	703.5 ± 18.8 ^b^	728.6 ± 12.2 ^b^	<0.0001	0.09	<0.0001
Insulin AUC, mmol/L/120 min	169.5 ± 22.8 ^c^	226.8 ± 6.2 ^bc^	228.7 ± 13.9 ^bc^	391.8 ± 16.2 ^a^	226.5 ± 18.3 ^bc^	254.5 ± 33.9 ^b^	<0.0001	0.03	<0.0001
Retroperitoneal fat, mg/mm tibial length	138.8 ± 11.1 ^b^	127.8 ± 10.6 ^b^	150.4 ± 9.3 ^b^	331.5 ± 20.9 ^a^	303.3 ± 16.9 ^a^	322.0 ± 18.8 ^a^	<0.0001	0.32	0.76
Epididymal fat, mg/mm tibial length	105.3 ± 5.7 ^b^	91.3 ± 4.8 ^b^	115.3 ± 8.4 ^b^	170.4 ± 11.4 ^a^	200.3 ± 15.4 ^a^	197.1 ± 16.7 ^a^	<0.0001	0.26	0.15
Omental fat, mg/mm tibial length	59.7 ± 5.7 ^b^	63.1 ± 4.6 ^b^	74.3 ± 6.6 ^b^	131.6 ± 10.6 ^a^	125.4 ± 12.3 ^a^	115.3 ± 14.4 ^a^	<0.0001	0.99	0.27
Total abdominal fat, mg/mm tibial length	292.7 ± 19.6 ^b^	268.9 ± 26.0 ^b^	340.0 ± 23.1 ^b^	670.1 ± 46.9 ^a^	628.9 ± 42.4 ^a^	634.4 ± 40.2^ a^	<0.0001	0.49	0.45
Visceral adiposity index, %	3.7 ± 0.3 ^b^	3.0 ± 0.3 ^b^	3.9 ± 0.3 ^b^	6.9 ± 0.5 ^a^	7.0 ± 0.4 ^a^	7.3 ± 0.7 ^a^	<0.0001	0.41	0.65
Plasma NEFA, mmol/L	1.47 ± 0.18^ c^	1.46 ± 0.28^ c^	1.55 ± 0.09^ c^	2.78 ± 0.28^ b^	3.73 ± 0.29^ a^	2.72 ± 0.47^ b^	<0.0001	0.18	0.12
Plasma triglycerides, mmol/L	0.43 ± 0.07 ^c^	0.41 ± 0.07^ c^	0.54 ± 0.07^ c^	1.29 ± 0.19^ a^	1.17 ± 0.14^ ab^	0.80 ± 0.19^ bc^	<0.0001	0.38	0.07
Plasma total cholesterol, mmol/L	1.52 ± 0.09^ b^	1.51 ± 0.04^ b^	1.53 ± 0.05^ b^	1.98 ± 0.05^ a^	1.87 ± 0.19^ ab^	1.65 ± 0.09 ^b^	0.0002	0.26	0.20

Values are mean ± SEM, *n* = 8–10. Means within a row with the same superscript are not significantly different, *p* < 0.05. C, corn starch fed rats; CUO, corn starch rats treated with *Ulva ohnoi*; CDT, corn starch rats treated with *Derbesia tenuissima*; H, high-carbohydrate, high-fat diet fed rats; HUO, high-carbohydrate, high-fat rats treated with *Ulva ohnoi*; HDT, high-carbohydrate, high-fat rats treated with *Derbesia tenuissima*; AUC, area under curve.

**Table 3:** Cardiovascular parameters in rats fed C or H and with either *Ulva ohnoi* or *Derbesia tenuissima*.

Variable	C	CUO	CDT	H	HUO	HDT	*p*-Value		
							Diet	Treatment	Interaction
LV + septum, mg/mm tibial length	17.1 ± 0.3^ b^	18.9 ± 0.4^ ab^	20.5 ± 1.0^ a^	17.2 ± 0.5^ b^	17.3 ± 0.3^ b^	18.1 ± 1.0^ b^	0.023	0.010	0.18
RV wet weight, mg/mm tibial length	2.2 ± 0.2^ ab^	2.7 ± 0.2^ ab^	2.9 ± 0.4^ a^	2.2 ± 0.1^ ab^	2.0 ± 0.1^ ab^	1.9 ± 0.2^ b^	0.004	0.67	0.10
Heart wet weight, mg/mm tibial length	19.3 ± 0.4^ b^	20.5 ± 0.5^ b^	23.3 ± 1.4^ a^	19.4 ± 0.5^ b^	19.2 ± 0.5^ b^	19.3 ± 0.5^ b^	0.006	0.030	0.025
Systolic blood pressure, mmHg	127 ± 2 ^c^	126 ± 3 ^c^	131 ± 1 ^bc^	157 ± 1 ^a^	128 ± 1^bc^	135 ± 3 ^b^	<0.0001	<0.0001	<0.0001
SBP:LVIDs	35.2 ± 1.8 ^b^	29.4 ± 2.0 ^c^	30.4 ± 1.9 ^c^	47.3 ± 3.9^ a^	29.2 ± 0.9^ b^	40.1 ± 1.6 ^b^	0.0006	<0.0001	0.033
SBP:systolic volume	3096 ± 525 ^ab^	1731 ± 363 ^b^	1716 ± 340 ^b^	4818 ± 1129 ^a^	1497 ± 137^ b^	3690 ± 458 ^ab^	0.035	0.003	0.18
ESS:LVIDs	2.02 ± 0.07 ^b^	2.10 ± 0.06 ^b^	2.09 ± 0.08 ^b^	2.44 ± 0.09 ^a^	2.17 ± 0.07 ^b^	2.02 ± 0.08 ^b^	0.032	0.09	0.008
Diastolic stiffness *κ*	23.9 ± 1.7 ^b^	23.3 ± 0.4 ^b^	23.0 ± 0.6 ^b^	29.8 ± 2.2 ^a^	25.1 ± 0.9 ^b^	23.4 ± 1.0 ^b^	0.016	0.023	0.11

Values are mean ± SEM, *n* = 8–10. Means within a row with the same superscript are not significantly different, *p* < 0.05. C, corn starch fed rats; CUO, corn starch rats treated with *Ulva ohnoi*; CDT, corn starch rats treated with *Derbesia tenuissima*; H, high-carbohydrate, high-fat diet fed rats; HUO, high-carbohydrate, high-fat rats treated with *Ulva ohnoi*; HDT, high-carbohydrate, high-fat rats treated with *Derbesia tenuissima*; SBP, systolic blood pressure; LV, left ventricle; RV right ventricle; LVIDs, left ventricular internal diameter in systole; ESS, end-systolic stress.

**Table 4:** Liver parameters and plasma biochemistry in rats fed C or H and with either *Ulva ohnoi* or *Derbesia tenuissima*.

Variable	C	CUO	CDT	H	HUO	HDT	*p*-Value		
							Diet	Treatment	Interaction
Liver weight	215.7 ± 6.8^ b^	236.6 ± 13.9^ b^	229.9 ± 7.9^ b^	277.5 ± 13.6^ a^	301.2 ± 10.2^ a^	269.3 ± 18.0^ a^	<0.0001	0.17	0.55
Plasma ALT activity, U/L	28.2 ± 2.4^ b^	32.4 ± 1.8^ b^	36.5 ± 3.1^ b^	47.4 ± 4.1^ a^	30.2 ± 2.6^ b^	31.9 ± 2.9^ b^	0.26	0.10	0.0002
Plasma AST activity, U/L	71.7 ± 5.3^ b^	84.1 ± 2.6^ b^	85.5 ± 3.3^ b^	102.4 ± 3.4^ a^	70.8 ± 4.5^ b^	89.5 ± 3.5^ b^	0.10	0.05	<0.0001
Plasma Na^+^, mmol/L	143 ± 1	143 ± 1	141 ± 1	143 ± 0	140 ± 1	140 ± 1	0.07	0.03	0.24
Plasma K^+^, mmol/L	5.55 ± 0.38	5.94 ± 0.33	6.43 ± 0.31	6.03 ± 0.64	6.75 ± 1.04	5.20 ± 0.48	0.97	0.57	0.19
Plasma Mg^2+^, mmol/L	0.78 ± 0.03	0.86 ± 0.04	0.83 ± 0.02	0.79 ± 0.03	0.89 ± 0.04	0.81 ± 0.03	0.80	0.027	0.74

Values are mean ± SEM, *n* = 8–10. Means within a row with the same superscript are not statistically different, *P* < 0.05. C, corn starch fed rats; CUO, corn starch rats treated with *Ulva ohnoi*; CDT, corn starch rats treated with *Derbesia tenuissima*; H, high-carbohydrate, high-fat diet fed rats; HUO, high-carbohydrate, high-fat rats treated with *Ulva ohnoi*; HDT, high-carbohydrate, high-fat rats treated with *Derbesia tenuissima*; ALT, alanine transaminase; AST, aspartate transaminase.

**Figure 1:**
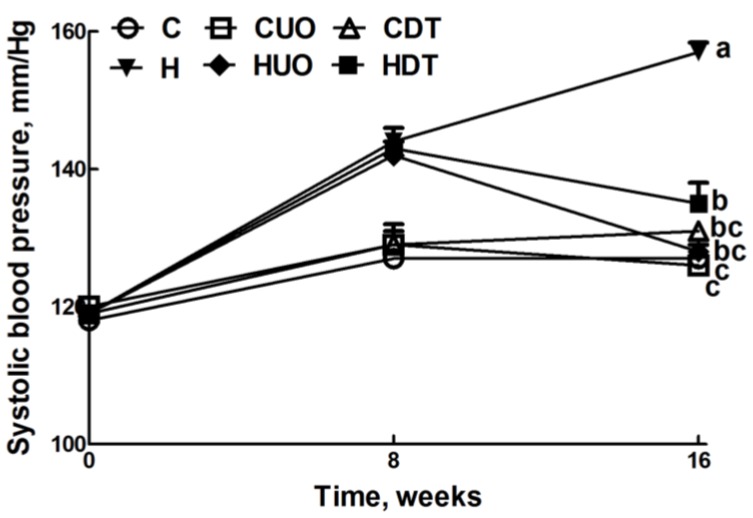
Effects of seaweeds treatment on systolic blood pressure. Values are mean ± SEM, *n* = 8–10. Endpoint means without a common letter differ, *p* < 0.05. C, corn starch fed rats; CUO, corn starch rats treated with *Ulva ohnoi*; CDT, corn starch rats treated with *Derbesia tenuissima*; H, high-carbohydrate, high-fat diet fed rats; HUO, high-carbohydrate, high-fat rats treated with *Ulva ohnoi*; HDT, high-carbohydrate, high-fat rats treated with *Derbesia tenuissima*.

### 2.3. Cardiovascular Structure and Function

**Figure 2:**
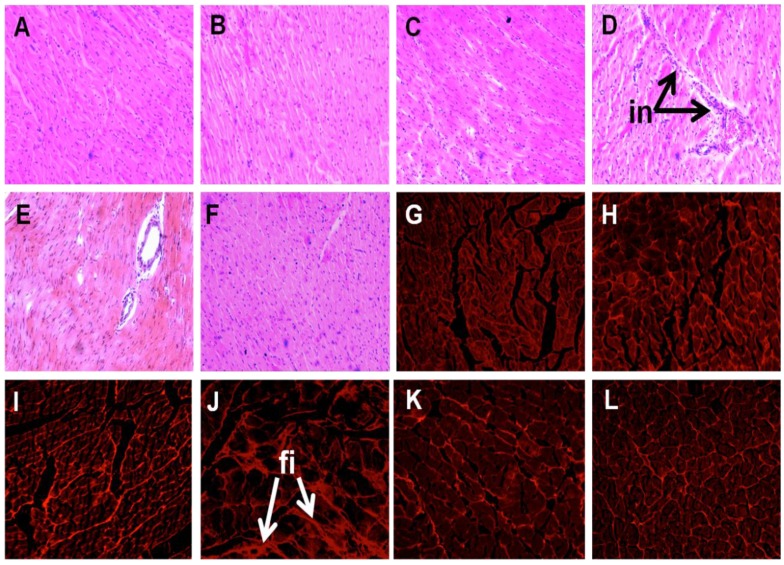
Effects of seaweeds treatment on inflammation and fibrosis in the heart. Haematoxylin and eosin staining of left ventricle showing infiltration of inflammatory cells (**A**–**F**, inflammatory cells marked as “in”) (×20) from C (**A**); CUO (**B**); CDT (**C**); H (**D**); HUO (**E**) and HDT (**F**) rats. Picrosirius red staining of left ventricle showing collagen deposition (**G**–**M**, fibrosis marked as “fi”)(20×) from C (**A**); CUO (**B**); CDT (**C**); H (**D**); HUO (**E**) and HDT (**F**) rats. C, corn starch fed rats; CUO, corn starch rats treated with *Ulva ohnoi*; CDT, corn starch rats treated with *Derbesia tenuissima*; H, high-carbohydrate, high-fat diet fed rats; HUO, high-carbohydrate, high-fat rats treated with *Ulva ohnoi*; HDT, high carbohydrate, high fat rats treated with *Derbesia tenuissima*.

Cardiovascular parameters are given in Table 3. Systolic blood pressure was unchanged in C groups (Figure 1). The higher systolic blood pressure in H rats was lowered in HUO and HDT rats (Figure 1). The ratios of SBP:LVIDs and ESS:LVIDs were higher in H rats compared to C groups, while no change was observed in SBP:systolic volume ratio. Ventricular contractility estimated as SBP:LVIDs and ESS:LVIDs was also normalised in HUO and HDT rats. No changes in other echocardiographic parameters were observed in the seaweed treatment groups compared to H and C groups (Supplementary Table S4). The left ventricle showed increased infiltration of inflammatory cells (Figure 2D) and collagen deposition (Figure 2J) in H rats, compared with C groups (Figure 2A–C,G–I). In HUO and HDT rats, the infiltration of inflammatory cells (Figure 2E,F) and the interstitial collagen deposition (Figure 2K,L) were normalised. The diastolic stiffness constant (*κ*) was normalised in HUO and HDT rats, compared to H rats.

**Figure 3:**
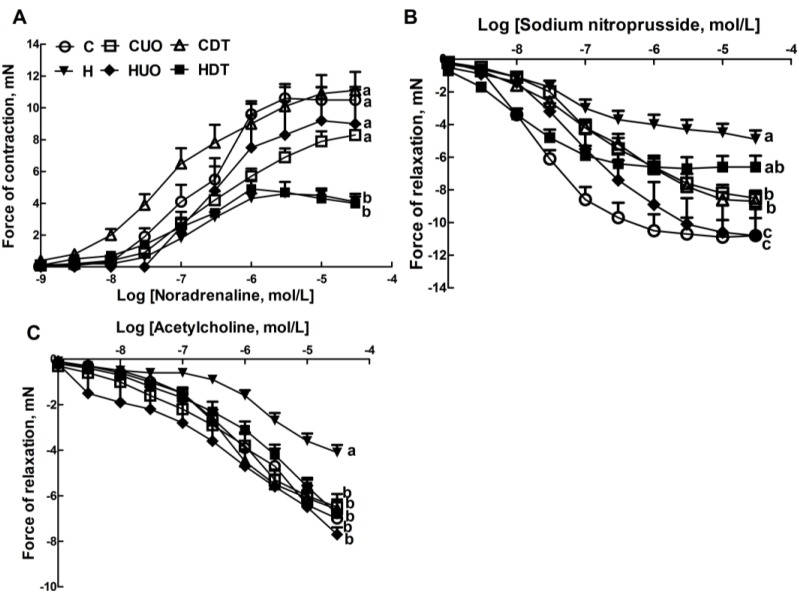
Effects of seaweeds treatment on noradrenaline-induced contraction (**A**); sodium nitroprusside-induced relaxation (**B**); and acetylcholine-induced relaxation (**C**) in thoracic aortic preparations from C, CUO, CDT, H, HUO and HDT rats. Values are mean ± SEM, *n* = 8–10. Endpoint means without a common letter differ, *p* < 0.05. C, corn starch fed rats; CUO, corn starch rats treated with *Ulva ohnoi*; CDT, corn starch rats treated with *Derbesia tenuissima*; H, high-carbohydrate, high-fat diet fed rats; HUO, high-carbohydrate, high-fat rats treated with *Ulva ohnoi*; HDT, high-carbohydrate, high-fat rats treated with *Derbesia tenuissima*.

Lower contractile responses to noradrenaline in isolated thoracic rings were measured in H and HDT rats compared to C and HUO rats (Figure 3A). H rats showed lower smooth muscle-dependent and endothelium-dependent relaxant responses to sodium nitroprusside and acetylcholine (Figure 3B,C); both responses were higher in HUO rats and acetylcholine responses were higher in HDT rats (Figure 3B,C).

### 2.4. Liver Structure and Function

**Figure 4:**
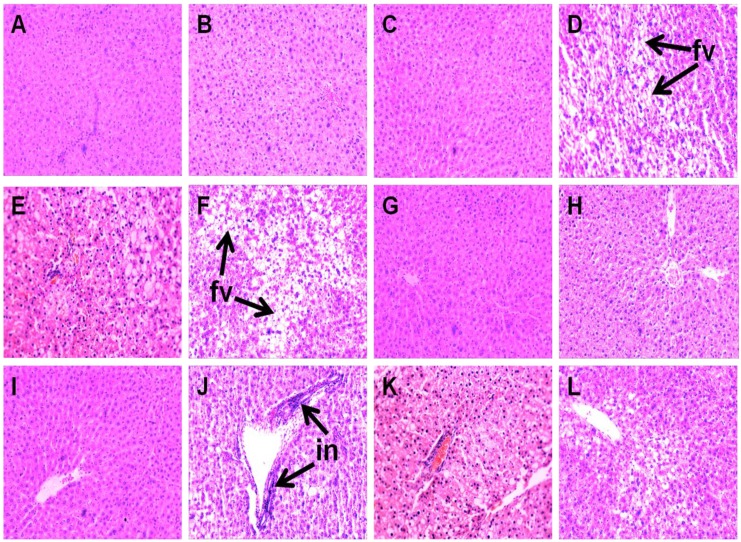
Effects of seaweeds treatment on inflammation and fat deposition in the liver. Haematoxylin and eosin staining of liver showing enlarged fat vacuoles (**A**–**F**, marked as “fv”) (×20) and inflammatory cells (**G**–**L**, marked as “in”) (×20) from C (**A**,**G**); CUO (**B**,**H**); CDT (**C**,**I**); H (**D**,**J**); HUO (**E**,**K**) and HDT (**F**,**L**) rats. C, corn starch fed rats; CUO, corn starch rats treated with *Ulva ohnoi*; CDT, corn starch rats treated with *Derbesia tenuissima*; H, high-carbohydrate, high-fat diet fed rats; HUO, high-carbohydrate, high-fat rats treated with *Ulva ohnoi*; HDT, high-carbohydrate, high-fat rats treated with *Derbesia tenuissima*.

Liver parameters are given in Table 4. Compared to C groups, H rats showed increased liver weight, higher infiltration of inflammatory cells and presence of enlarged fat vacuoles (Figure 4A–D,G–J). UO and DT treatment prevented the infiltration of inflammatory cells in HUO or HDT rats (Figure 4K,L). Liver weight was unchanged in HUO or HDT rats compared to H rats. However, hepatocytes with enlarged fat vacuoles were observed in HDT rats but absent in HUO rats (Figure 4E,F). Plasma activities of liver enzymes alanine transaminase (ALT) and aspartate transaminase (AST) were higher in H rats compared to C treatment groups whereas both the ALT and AST activities were normalised in HUO and HDT rats.

## 3. Discussion

The prevalence of obesity is increasing in westernised populations, largely due to consumption of food that is rich in refined carbohydrates, omega-6 fatty acids, saturated and mono-unsaturated fatty acids [1,2]. In eastern Asia, seaweeds are acknowledged as functional foods, referring to the presence of nutrients that may reduce potential damage from chronic diseases [6,8], but they are not widely recognised as functional foods in westernised populations. In this study, we have demonstrated the ability of two tropical seaweeds that can be grown commercially, *Ulva ohnoi* (UO) and *Derbesia tenuissima* (DT), to attenuate or normalise a range of metabolic syndromes in rats that were induced using a diet with increased simple sugars and saturated fats [20,21,22]. *Ulva* represents a group of seaweeds present in all oceans, from tropical to temperate waters, that are highly suited to intensive aquaculture production [17]. *Derbesia* has been identified as a target group for functional food and intensive aquaculture production based on its unique nutritional and biochemical characteristics [18]. DT contains omega-3 polyunsaturated fatty acids including eicosapentaenoic acid and α-linolenic acid with a higher fat content than UO [13,18]. However, the intakes of α-linolenic acid (HUO:5.6 mg/day; HDT:16.6 mg/day) and eicosapentaenoic acid (HUO:0.0 mg/day; HDT:1.3 mg/day) in this study are much lower than the intake of α-linolenic acid (350 mg/day) and eicosapentaenoic acid (550 mg/day) that attenuated diet-induced metabolic syndrome in previous studies using the same model [22]. Thus, the intakes of omega-3 polyunsaturated fatty acids in this study are unlikely to explain the differences between seaweeds or be sufficient on their own to reverse metabolic changes in the H rats. Therefore, we now focus on three possible mechanisms whereby increased intake of fibre or magnesium ions could improve metabolic syndrome by acting as prebiotics, improving cardiovascular function or decreasing inflammation.

Differing fibre contents with UO containing similar amounts of both soluble and insoluble fibre and DT containing only insoluble fibre could partially explain the different responses to these two seaweeds. Dietary fibre, including both soluble (ß-glucans, pectins, gums, mucilages and some hemicelluloses) and insoluble fibre (celluloses and hemicelluloses) from whole grains, vegetables and fruits, was directly associated with reducing the risk factors of cardiovascular disease, and the management of obesity, hypertension, hyperlipidaemia and diabetes [23]. The American Dietetic Association recommends a daily dietary fibre intake of 25 g for adult females and 38 g for adult men [24]. Based on body surface area, conversion of the seaweed intake in the current study in rats to humans [25] would provide a fibre intake of between 5.8 and 15.6 g/day, lower than the recommendations, but sufficient to double current intake in the USA. The physicochemical properties of seaweed fibre, such as the ability to absorb and hold water, ion exchange capacity and viscosity, will slow down absorption of compounds in the gut [26,27]. We suggest that the high soluble fibre content of UO could increase gastrointestinal viscosity and therefore inhibit intestinal absorption of fatty acids more effectively than DT, leading to the decreased total body fat mass with UO but not DT. Both UO and DT treatments improved glucose utilisation and insulin sensitivity, potentially through similar mechanisms. This has been shown with viscous soluble dietary fibre from linseed in young male Wistar rats [28]. Further, the fibre present in green seaweed is more similar to terrestrial crops than the fibre present in red and brown seaweed [29]. The fibre may work as a prebiotic, defined as polysaccharides that are not broken down in the stomach but are fermented in the intestine to improve gut bacteria [30]; these changes in intestinal bacteria could prevent obesity [31].

In addition to fibre, the mineral ion contents of the seaweed supplements were up to 3-fold higher than the control diets. The increased magnesium content in UO could delay the onset of diabetes as reported in OLETF rats where an increased magnesium intake of 16 mg/day [32] improved insulin sensitivity and glucose utilisation [33]. In contrast, magnesium deficiency aggravated the insulin resistance produced by high fat diets given to growing rats [34]. None of the other major minerals in the seaweeds such as potassium, calcium and zinc were present in sufficient quantities to have bioactive effects on the metabolic syndrome [35,36,37].

The potential influence of fibre and minerals on metabolic syndrome extended to other physiological changes in the high-carbohydrate, high-fat fed rats with attenuation or normalisation of the increases in blood pressure, ventricular diastolic stiffness, fibrosis and liver damage. High dietary fibre supplements in hypertensive subjects reduced systolic and diastolic blood pressures compared to subjects with low dietary fibre [38]. Increased dietary fibre may reduce blood pressure by reducing insulin resistance, by reducing fat stores [6,39] or by increasing magnesium intake [33], or all three as in this study. The decreased infiltration of inflammatory cells probably precedes the decreased collagen synthesis and deposition in both the heart and liver. Both UO and DT supplementation improved liver structure and function, with multiple potential mechanisms such as improved insulin sensitivity and glucose tolerance, decreased blood pressure, decreased fat deposition and decreased infiltration of inflammatory cells. Further, supplementation of DT to H rats reduced the plasma concentrations of triglycerides and total cholesterol. We suggest that the bioactive polysaccharides present in DT and UO may be responsible for these cardiovascular and liver responses [6,9] and deserve further study.

Inflammation is critically important in the development of obesity [40]; we showed increased infiltration of inflammatory cells in the heart and liver of H rats that was markedly attenuated by both UO and DT. In human adults with an increased fat intake of 78.8–84.2 g/day, increased dietary fibre intake of 14.3–16.6 g/day decreased serum C-reactive protein concentrations, a non-specific marker of inflammation [41]. Bioactive polysaccharides from many different seaweeds have demonstrated anti-inflammatory activity [42]. Furthermore,* in vivo* and* in vitro* studies observed that dietary fibre and short-chain carboxylic acids, such as propionate and butyrate, released during the bacterial fermentation of dietary fibre as prebiotics attenuated the production of pro-inflammatory cytokines including interleukin-8, interleukin-6 and tumour necrosis factor-α [43]. Similar modes of action are likely with green seaweed polysaccharides [29].

## 4. Experimental Section

### 4.1. Resources: Algae, Diet Components, Rats

Two species of green algae (Chlorophyta), *Ulva ohnoi* (UO) and *Derbesia tenuissima* (DT), were cultured in the aquaculture facilities of James Cook University, Townsville, Australia. All biomass was produced in large outdoor tanks with capacities of 2500 L for *Derbesia* and >10,000 L for *Ulva*. Biomass was harvested on two occasions separated by ~12 months in 2011–2012. The biomass was rinsed in freshwater and freeze-dried, after which sub-samples were taken for analyses of fibre components (100 g dry-weight), and minerals and fatty acid concentrations (200 mg dry-weight).

In each sub-sample, 24 trace elements were quantified and mean values are reported (*n* = 2 sub-samples per species). Al, Ca, K, Na, S and P were analysed by Inductively Coupled Plasma Optical Emission Spectrometry, while metals and metalloids (As, B, Ba, Cd, Co, Cr, Cu, Fe, Hg, Mg, Mn, Mo, Ni, Pb, Se, Sr, V and Zn) were analysed by Inductively Coupled Plasma Mass Spectrometry at the Advanced Analytical Centre, James Cook University, Townsville. Insoluble and soluble fibre was analysed on a combined sample of 100 g for each species containing 50 g from each harvest time. Fibre analyses were run using enzymatic-gravimetric methods by National Measurement Institute, Sydney, Australia (AOAC Official Method 985.29 for insoluble fibre; AOAC Official Method 993.19 for soluble fibre). Total crude lipids and fatty acids were extracted and analysed [13]. All remaining freeze-dried biomass was stored in vacuum-sealed bags under refrigeration until preparation of the food.

The experimental groups consisted of 72 male Wistar rats (9–10 weeks-old; 336 ± 2 g) individually housed in a temperature-controlled (20 ± 2 °C), 12 h light/dark cycle environment with unrestricted access to water and food. The CS diet contained 570 g cornstarch, 155 g powdered rat food (Specialty Feeds, Glen Forest, WA, Australia), 25 g Hubble, Mendel and Wakeman (HMW) salt mixture and 250 g water per kilogram of diet. The HCHF diet consisted of 175 g fructose, 395 g sweetened condensed milk, 200 g beef tallow, 155 g powdered rat food, 25 g HMW salt mixture and 50 g water per kilogram of diet. In addition, the drinking water for the HCHF group was supplemented with 25% fructose. Energy intake was calculated from the following values in kJ/g: fructose, 15.40; corn starch, 15.94; condensed milk, 13.80; beef tallow, 37.70 and powdered rat food, 13.80. The energy densities of the CS diet and the HCHF diet were 11.23 kJ/g and 17.83 kJ/g of food respectively and an additional 3.85 kJ/mL in the drinking water for the HCHF diet-fed rats [20,21]. The rats were randomly divided into 6 separate groups (*n* = 12 each) and fed with corn starch (C), corn starch + *Ulva ohnoi* 5% (CUO), corn starch + *Derbesia tenuissima* 5% (CDT), high-carbohydrate, high-fat (H), high-carbohydrate, high-fat + *Ulva ohnoi* 5% (HUO), or high-carbohydrate, high-fat + *Derbesia tenuissima* 5% (HDT). The seaweed-supplemented diets were prepared by adding 5% of seaweeds to replace an equivalent amount of water in the diet. The drinking water in all H groups included 25% fructose. The seaweed-supplemented diets were administered for 8 weeks starting 8 weeks after the initiation of the C or H diets.

All experimentation was approved by the Animal Ethics Committees of The University of Queensland and University of Southern Queensland under the guidelines of the National Health and Medical Research Council of Australia. Rats were monitored daily for body weight, and food and water intakes. Daily seaweed intake was calculated from the daily food intake. The fatty acid concentrations of both C and H control diets used for the calculation of mean daily fatty acids intake were obtained from our previous study [22].

Abdominal circumference of rats was measured using a standard measuring tape during the period of anaesthesia for systolic blood pressure (SBP) measurements. SBP measurements, and oral glucose and insulin tolerance tests were conducted at 0, 8 and 16 weeks. Oral glucose tolerance tests were performed following determination of overnight fasting blood glucose concentrations in tail vein blood using Medisense Precision Q.I.D glucose meters (Abbott Laboratories, Bedford, MA, USA). For overnight fasting, rats were deprived of food for 12 h. Fructose-supplemented drinking water in HCHF groups was replaced with normal drinking water for the overnight food-deprivation period. Rats were given a glucose load of 2 g/kg body weight as 40% glucose solution via oral gavage and blood glucose concentrations were measured again 30, 60, 90 and 120 min after oral glucose administration. For insulin tolerance, basal blood glucose concentrations were measured after 4–5 h of food deprivation as above. The rats were injected ip 0.33 IU/kg insulin-R (Eli Lilly Australia, West Ryde, NSW, Australia), and tail vein blood samples were taken at 0, 30, 60, 90 and 120 min. Rats were withdrawn from the test if the blood glucose dropped below 1.1 mmol/L, and 4 g/kg glucose was immediately administered by oral gavage to prevent hypoglycemia [20,21,22]. Dual energy X-ray absorptiometric (DXA) measurements were performed on rats after 16 weeks of feeding, 2 days before euthanasia using a Norland XR36 DXA instrument (Norland Corp, Fort Atkinson, WI, USA). DXA scans were analysed using the manufacturer’s recommended software for use in laboratory animals (Small Subject Analysis Software, version 2.5.3/1.3.1; Norland Corp). The precision error of lean mass for replicate measurements, with repositioning, was 3.2% [20,21,22]. Feed conversion efficiency was calculated as [mean body weight gain (in grams)/daily energy intake (in kilojoules)]. Visceral adiposity index (%) was calculated [20,21,22].

**Figure 5:**
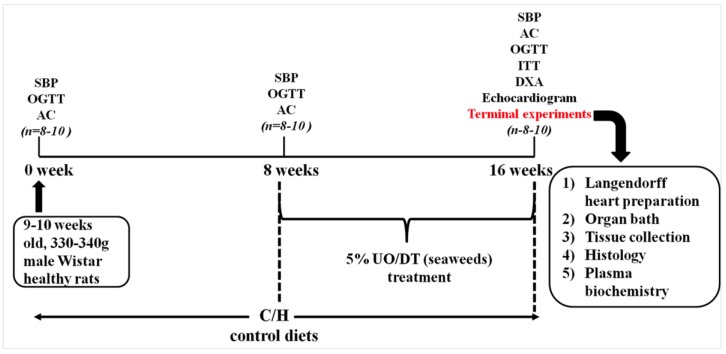
Outline of the experimental protocol for measurements on rats. SBP, systolic blood pressure; OGTT, oral glucose tolerance test; AC, abdominal circumference, ITT, insulin tolerance test; DEXA, dual energy X-ray absorptiometry; UO, *Ulva ohnoi*; DT, *Derbesia tenuissima*.

The experimental protocol is summarised in Figure 5.

### 4.2. Cardiovascular Measurements

Systolic blood pressure was measured under light sedation following intraperitoneal injection of Zoletil (tiletamine 15 mg/kg, zolazepam 15 mg/kg; Virbac, Milperra, Australia) using a MLT1010 Piezo-Electric Pulse Transducer (ADInstruments, Sydney, Australia) and inflatable tail-cuff connected to a MLT844 Physiological Pressure Transducer (ADInstruments, Sydney, Australia) using PowerLab data acquisition unit (ADInstruments, Sydney, Australia) [20,21,22].

Echocardiographic examinations (Phillips iE33, 12MHz transducer, Best, The Netherlands) were performed to assess the cardiovascular structure and function in all rats at 16 weeks [20,21,22]. Rats were anaesthetised using Zoletil (tiletamine 25 mg/kg and zolazepam 25 mg/kg, intraperitoneally (i.p); Virbac, Peakhurst, Australia) and Ilium Xylazil (xylazine 15 mg/kg, i.p.; Troy Laboratories, Smithfield, Australia). The ventricular contractility indexes were calculated including ratio of systolic blood pressure (SBP) to left ventricular internal diameter in systole (LVIDs), ratio of SBP to systolic volume, and ratio of end-systolic stress (ESS) to LVIDs [21,44].

The left ventricular function of the rats was assessed using the Langendorff heart preparation [20,21,22]. Terminal anaesthesia was induced via i.p. injection of pentobarbitone sodium (Lethabarb^®^, 100 mg/kg, Virbac, Milperra, Australia). Blood (~5 mL) was taken from the abdominal aorta after heparin (200 IU; Sigma-Aldrich Australia, Sydney, Australia) administration through the right femoral vein. Isovolumetric ventricular function was measured by inserting a latex balloon catheter into the left ventricle of the isolated heart connected to a Capto SP844 MLT844 physiological pressure transducer and Chart software on a MacLab system (ADInstruments, Sydney, Australia).

Thoracic aortic rings (~4 mm in length) were suspended in an organ bath chamber with a resting tension of approximately 10 mN. Cumulative concentration-response (contraction) curves were measured for noradrenaline (Sigma-Aldrich Australia, Sydney, Australia); concentration-response (relaxation) curves were measured for acetylcholine (Sigma-Aldrich Australia, Sydney, Australia) and sodium nitroprusside (Sigma-Aldrich Australia, Sydney, Australia) in the presence of a submaximal (70%) contraction to noradrenaline [20,21,22].

### 4.3. Organ Weights

The right and left ventricles were separated after Langendorff experiments and weighed. Liver, and retroperitoneal, epididymal and omental fat pads were removed following heart removal and blotted dry for weighing. Organ weights were normalised relative to the tibial length at the time of their removal (in mg/mm) [20,21,22].

### 4.4. Histology

Two rats per group were taken exclusively for histological analysis. Two slides were prepared per tissue specimen and two random, non-overlapping fields per slide were photographed. Immediately after removal, heart and liver tissues were fixed in 10% neutral buffered formalin for 3 days and then dehydrated and embedded in paraffin wax [20,21,22]. Thin sections (7 μm) of left ventricle and the liver were cut and stained with haematoxylin and eosin stain for determination of inflammatory cell infiltration with 20X and fat vacuole enlargement with 40× objectives using a Olympus BX51 microscope (Olympus, Melville, NY, USA). Collagen distribution was measured in the left ventricle with picrosirius red stain. Laser confocal microscopy (Zeiss LSM 510 upright Confocal Microscope, Carl Zeiss, North Ryde, Australia) was used to visualize collagen deposition [20,21,22].

### 4.5. Plasma Biochemistry

Blood was centrifuged within 30 min of collection into heparinised tubes at 5000 *g* for 15 min. Plasma was separated and transferred to Eppendorf tubes (Thermo Fisher Scientific, Scoresby, Australia) for storage at −20 °C before analysis. Activities of plasma enzymes and analyte concentrations were determined using kits and controls supplied by Olympus using an Olympus analyser (AU 400, Tokyo, Japan) [20,21,22].

### 4.6. Statistical Analysis

All data are presented as mean ± SEM. Data from C, CUO, CDT, H, HUO and HDT groups were compared in a series of two-way ANOVAs (Analysis of Variance) with two types of “Diet”, high carbohydrate and high fat diet or control cornstarch diet, and three types of “Treatment”, a control and treatments supplemented with each seaweed, as the two fixed factors in the analyses; C was compared with H, CUO or CDT; H was compared with HUO or HDT. Homogeneity of variance for ANOVA was assessed using Bartlett’s test and variables that were not normally distributed were log-transformed prior to analysis. Where the main effects were significant (*p* < 0.05), means were compared using Newman-Keuls multiple comparisons. Where transformations did not result in normality or homogeneity of variance, a Kruskal-Wallis non-parametric test was performed. All statistical analyses were run using GraphPad Prism version 5.00 for Windows (GraphPad Software, La Jolla, CA, USA).

## 5. Conclusions

Our findings suggest that soluble dietary fibre as a major component in UO could play a key role in attenuating the signs of the metabolic syndrome such as hypertension, endothelial dysfunction, diminished insulin sensitivity and glucose utilisation, increased cardiac stiffness, increased collagen deposition, increased liver damage and increased fat mass in diet-induced obese rats. Further, we suggest that the insoluble fibre of both DT and UO may improve glucose metabolism and that the increased magnesium intake in UO could delay the onset of diabetes. Thus, these tropical seaweeds serve as a commercially viable source of dietary fibre as a functional food, as they can be produced in large quantities by aquaculture to attenuate the signs of metabolic syndrome.

## Supplementary Files

Supplementary File 1
