# Supplementary material for: Seaweed Supplements Normalise Metabolic, Cardiovascular and Liver Responses in High-Carbohydrate, High-Fat Fed Rats
Source: Mar Drugs. 2015 Feb 2;13(2):788–805. doi: 10.3390/md13020788 (PMC4344602; doi:10.3390/md13020788)
Supplement: Supplementary File 1 [file marinedrugs-13-00788-s001.pdf]

Supplementary Information

Table S1. Micronutrient profile of diets.

Mineral Ions	Corn Starch (mg/kg)	High-Carbohydrate, High-Fat Diet (mg/kg)	*Ulva ohnoi* (mg/kg)	*Derbesia tenuissima* (mg/kg)
K	4760	4450	29,350	13,400
Na	1475	1335	27,650	61,100
Mg	914	689	41,300	13,000
Ca	7190	7790	2985	4870
Fe	256.5	185	58.5	360
P	2910	2500	742	3620
Mn	23.5	18.2	13.0	29.9
B	3.4	2.9	85.9	78.0
Al	17.6	20.9	16.3	13.7
Sr	14.0	12.4	36.3	77.2
Zn	22.1	18.3	12.0	36.3
Cu	8.6	7.1	11.7	16.9
Ni	0.4	0.3	2.8	2.0
Mo	0.3	0.1	0.3	0.4
V	3.3	5.3	1.0	1.4
As	1.4	1.2	0.0	4.3
Cr	10.0	16.0	1.8	3.2
Pb	≤0.05	≤0.05	0.3	0.9
Se	≤1	≤1	1.3	3.1
Co	≤0.1	≤0.1	0.3	0.5
Hg	≤0.5	≤0.5	1.3	4.4
Cd	≤0.05	≤0.05	0.2	0.3
**Sum total**	**18,291.0**	**18,049.2**	**134,477.1**	**114,723.4**

Mineral ions profile (mean of samples, $n = 2$).

Table S2. Essential mineral ion intake in rats fed control diets C or H and with either *Ulva ohnoi* or *Derbesia tenuissima*.

Variable	C	CUO	CDT	H	HUO	HDT	*p*-Value		
							Diet	Treatment	Interaction
Magnesium intake, mg/day	32.6 ± 3.3 [c]	97.8 ± 7.8 [a]	50.4 ± 3.8 [b]	15.1 ± 1.3 [d]	59.5 ± 5.1 [b]	29.3 ± 1.8 [c]	<0.0001	<0.0001	0.05
Potassium intake, mg/day	169.9 ± 17.4 [a]	204.5 ± 16.3 [a]	174.9 ± 13.0 [a]	97.2 ± 8.2 [b]	127.9 ± 10.9 [b]	112.0 ± 6.7 [b]	<0.0001	0.05	0.87
Sodium intake, mg/day	52.7 ± 5.4 [c]	93.9 ± 7.5 [b]	146.0 ± 10.9 [a]	29.2 ± 2.5 [d]	58.7 ± 5.0 [c]	96.0 ± 5.8 [b]	<0.0001	<0.0001	0.17
Calcium intake, mg/day	256.7 ± 26.3 [a]	241.1 ± 19.3 [ab]	239.5 ± 17.8 [ab]	170.1 ± 14.4 [b]	171.6 ± 14.6 [b]	175.7 ± 10.5 [b]	<0.0001	0.92	0.81
Zinc intake, mg/day	0.79 ± 0.08 [a]	0.75 ± 0.06 [a]	0.77 ± 0.06 [a]	0.40 ± 0.03 [b]	0.41 ± 0.03 [b]	0.44 ± 0.03 [b]	<0.0001	0.90	0.84

Values are mean ± SEM, n = 8–10, Mean within a row with the same superscript are not statistically different, $p < 0.05$. C, corn starch fed rats; CUO, cornstarch rats treated with *Ulva ohnoi*; CDT, cornstarch rats treated with *Derbesia tenuissima*; H, high-carbohydrate, high-fat diet fed rats; HUO, high-carbohydrate, high-fat rats treated with *Ulva ohnoi*; HDT, high-carbohydrate, high-fat rats treated with *Derbesia tenuissima*.

Table S3. Total fat, SF, MUFA and PUFA intake in rats fed control diets C or H and with either *Ulva ohnoi* or *Derbesia tenuissima*.

Variable	C	CUO	CDT	H	HUO	HDT	*p*-Value		
							Diet	Treatment	Interaction
Total fat intake, g/day (n = 8–10)	0.29 ± 0.02 [b]	0.28 ± 0.02 [b]	0.34 ± 0.03 [b]	5.23 ± 0.44 [a]	5.19 ± 0.44 [a]	5.29 ± 0.32 [a]	<0.0001	0.96	0.99
Saturated fatty acid, g/day (n = 8–10)	0.08 ± 0.01 [b]	0.09 ± 0.01 [b]	0.10 ± 0.00 [b]	2.77 ± 0.23 [a]	2.75 ± 0.23 [a]	2.80 ± 0.17 [a]	<0.0001	0.98	0.99
MUFA, g/day (n = 8–10)	0.10 ± 0.01 [b]	0.09 ± 0.01 [b]	0.10 ± 0.01 [b]	2.28 ± 0.19 [a]	2.29 ± 0.19 [a]	2.31 ± 0.14 [a]	<0.0001	0.99	0.99
PUFA, g/day (n = 8–10)	0.10 ± 0.01 [b]	0.09 ± 0.01 [b]	0.10 ± 0.01 [b]	0.15 ± 0.01 [a]	0.15 ± 0.01 [a]	0.17 ± 0.01 [a]	<0.0001	0.32	0.61
ALA, mg/day (n = 8–10)	13.2 ± 1.4 [c]	21.4 ± 1.7 [b]	28.9 ± 2.2 [a]	5.0 ± 0.0 [d]	5.6 ± 0.5 [d]	16.6 ± 0.9 [c]	<0.0001	<0.0001	0.03
EPA, mg/day (n = 8–10)	0.0 ± 0.0 [c]	0.0 ± 0.0 [c]	1.9 ± 0.1 [a]	0.0 ± 0.0 [c]	0.0 ± 0.0 [c]	1.3 ± 0.1 [b]	0.0002	<0.0001	<0.0001

Values are mean ± SEM, n = 8–10. Means within a row with the same superscript are not statistically different, $p < 0.05$. C, corn starch fed rats; CUO, cornstarch rats treated with *Ulva ohnoi*; CDT, cornstarch rats treated with *Derbesia tenuissima*; H, high-carbohydrate, high-fat diet fed rats; HUO, high-carbohydrate, high-fat rats treated with *Ulva ohnoi*; HDT, high-carbohydrate, high-fat rats treated with *Derbesia tenuissima*; [2] MUFA, mono-unsaturated fatty acid; PUFA, polyunsaturated fatty acid; ALA, alpha-linolenic acid; EPA, eicosapentaenoic acid.

Table S4. Cardiovascular structure and function in rats fed control diets C or H and with either *Ulva ohnoi* or *Derbesia tenuissima*.

Variable	C	CUO	CDT	H	HUO	HDT	*p*-Value		
							**Diet**	**Treatment**	**Interaction**
LVIDd, mm	7.3 ± 0.2	7.7 ± 0.2	7.8 ± 0.2	7.7 ± 0.2	7.9 ± 0.1	7.7 ± 0.4	0.38	0.39	0.56
LVIDs, mm	3.1 ± 0.3 [b]	4.3 ± 0.3 [a]	4.4 ± 0.3 [a]	3.5 ± 0.3 [b]	4.4 ± 0.1 [a]	3.3 ± 0.1 [b]	0.36	0.001	0.016
Heart rate	332.7 ± 33.1 [a]	252.3 ± 12.5 [b]	245.0 ± 6.8 [b]	373.0 ± 15.5 [a]	217.6 ± 5.1 [b]	393.6 ± 33.5 [a]	0.007	<0.0001	0.001
IVSd, mm	1.8 ± 0.0	1.9 ± 0.0	2.0 ± 0.1	1.9 ± 0.0	1.9 ± 0.0	1.9 ± 0.1	1.00	0.18	0.18
IVSs, mm	3.30 ± 0.09	3.00 ± 0.13	3.35 ± 0.10	3.39 ± 0.10	3.20 ± 0.09	3.44 ± 0.12	0.15	0.017	0.84
LVPWs, mm	3.00 ± 0.07	2.93 ± 0.09	3.15 ± 0.12	3.26 ± 0.13	2.95 ± 0.09	3.28 ± 0.11	0.12	0.036	0.52
LVPWd, mm	1.83 ± 0.06 [b]	1.76 ± 0.04 [b]	1.99 ± 0.04 [a]	1.85 ± 0.03 [ab]	1.85 ± 0.04 [ab]	1.83 ± 0.07 [ab]	0.68	0.104	0.040
Diastolic volume, μL	377.8 ± 35.7	463.0 ± 35.9	497.0 ± 31.5	482.0 ± 35.4	516.0 ± 21.6	485.0 ± 36.3	0.09	0.13	0.24
Systolic volume, μL	48.0 ± 6.3 [b]	88.0 ± 15.0 [a]	95.0 ± 15.4 [a]	53.0 ± 11.8 [b]	90.0 ± 7.4 [a]	40.0 ± 5.0 [b]	0.08	0.004	0.012
SBP:LVIDs	35.2 ± 1.8 [b]	29.4 ± 2.0 [c]	30.4 ± 1.9 [c]	47.3 ± 3.9 [a]	29.2 ± 0.9 [b]	40.1 ± 1.6 [b]	0.0006	<0.0001	0.033
SBP:systolic volume	3096 ± 525 [ab]	1731 ± 363 [b]	1716 ± 340 [b]	4818 ± 1129 [a]	1497 ± 137 [b]	3690 ± 458 [ab]	0.035	0.003	0.18
ESS:LVIDs	2.02 ± 0.07 [b]	2.10 ± 0.06 [b]	2.09 ± 0.08 [b]	2.44 ± 0.09 [a]	2.17 ± 0.07 [b]	2.02 ± 0.08 [b]	0.032	0.09	0.008
Stroke volume, μL	330.0 ± 33.9	375.0 ± 32.5	456.0 ± 44.7	429.0 ± 29.5	452.0 ± 25.3	445.0 ± 33.8	0.05	0.12	0.24
Cardiac output, mL/min	112.9 ± 20.1 [bc]	93.6 ± 7.4 [c]	128.7 ± 20.6 [abc]	159.5 ± 11.9 [ab]	98.1 ± 5.1 [c]	172.2 ± 16.7 [a]	0.016	0.003	0.32
Relative wall thickness	0.52 ± 0.02	0.49 ± 0.02	0.49 ± 0.03	0.47 ± 0.03	0.47 ± 0.01	0.49 ± 0.02	0.23	0.81	0.57
Systolic wall stress	71.4 ± 4.5	90.7 ± 6.8	92.1 ± 6.4	87.5 ± 9.4	95.9 ± 4.9	68.1 ± 5.4	0.48	0.12	0.035
Fractional shortening, %	50.1 ± 2.4 [ab]	49.8 ± 1.3 [ab]	47.1 ± 2.0 [ab]	51.3 ± 3.5 [ab]	45.0 ± 1.1 [b]	56.7 ± 1.4 [a]	0.30	0.14	0.012
Ejection fraction, %	86.9 ± 1.6 [ab]	81.2 ± 1.3 [b]	84.5 ± 1.8 [b]	87.8 ± 2.6 [ab]	83.5 ± 0.9 [b]	91.8 ± 0.8 [a]	0.018	0.003	0.17
Estimated LV mass, *g*	0.90 ± 0.10 [b]	1.04 ± 0.04 [b]	1.36 ± 0.12 [a]	1.03 ± 0.08 [b]	1.17 ± 0.03 [ab]	1.09 ± 0.06 [ab]	0.96	0.010	0.025

Values are mean ± SEM, n = 8–10. Means within a row with the same superscript are not statistically different, $p < 0.05$. C, corn starch fed rats; CUO, cornstarch rats treated with *Ulva ohnoi*; CDT, cornstarch rats treated with *Derbesia tenuissima*; H, high-carbohydrate, high-fat diet fed rats; HUO, high-carbohydrate, high-fat rats treated with *Ulva ohnoi*; HDT, high-carbohydrate, high-fat rats treated with *Derbesia tenuissima*; [2] LVIDd, left ventricular internal diameter during diastole; LVIDs, left ventricular internal diameter during systole; IVSd, interventricular septum during diastole; IVSs, interventricular septum thickness during systole; LVPWd, left ventricular posterior wall thickness during diastole; LVPWs, left ventricular posterior wall thickness during systole; SBP, systolic blood pressure; ESS, end systolic wall stress.

© 2015 by the authors; licensee MDPI, Basel, Switzerland. This article is an open access article distributed under the terms and conditions of the Creative Commons Attribution license (http://creativecommons.org/licenses/by/4.0/).
